# Genetic Differentiation and Origin of Naturalized Rainbow Trout Populations From Southern Chile, Revealed by the mtDNA Control Region Marker

**DOI:** 10.3389/fgene.2019.01212

**Published:** 2019-12-20

**Authors:** Nelson Colihueque, Francisco J. Estay, Julio E. Crespo, Aldo Arriagada, Luisa Baessolo, Cristian B. Canales-Aguirre, Javier Marín, René Carrasco

**Affiliations:** ^1^Laboratorio de Biología Molecular y Citogenética, Departamento de Ciencias Biológicas y Biodiversidad, Universidad de Los Lagos, Osorno, Chile; ^2^Gerencia de Investigación y Desarrollo, Piscícola Huililco Ltda., Pucón, Chile; ^3^Laboratorio de Ciencias Naturales y Sostenibilidad-Programa IBAM, Departamento de Ciencias Biológicas y Biodiversidad, Universidad de Los Lagos, Osorno, Chile; ^4^Colegio Proyección Siglo XXI, Osorno, Chile; ^5^Centro i∼mar, Universidad de Los Lagos, Puerto Montt, Chile; ^6^Núcleo Milenio de Salmónidos Invasores (INVASAL), Concepción, Chile; ^7^Programa de Pesca Recreativa, Departamento de Acuicultura y Recursos Agroalimentarios, Universidad de Los Lagos, Osorno, Chile

**Keywords:** rainbow trout, genetic-diversity, population genetics, origin, mtDNA control region, introduced species, self-sustaining populations, phylogenetic analysis

## Abstract

Numerous self-sustaining naturalized or introduced populations of rainbow trout (*Oncorhynchus mykiss*) are widely distributed throughout the freshwaters of southern Chile. In this study, analysis of the mitochondrial DNA control region (CR) marker was conducted to investigate the level of genetic divergence among populations and their phylogenetic relationships with respect to native lineages. This information provided a framework to interpret the genetic structure and origin that was shaped during historical trout introduction efforts. To this end, we analyzed eleven naturalized populations of lakes and rivers from five basins. The CR marker revealed five haplotypes. The overall haplotype (*H*) and nucleotide (*Π*) diversities were 0.684 ± 0.030 and 0.00460 ± 0.00012, respectively. Global *F* *_ST_* was 0.169, with several pairwise *F* *_ST_* estimates showing significant differences (*P* < 0.05). The exact test of population differentiation corroborated this result (*P* < 0.001). Significant geographic structure was found (*P* < 0.05), with variations explained primarily by differences within populations (61.65%) and among group basins (20.82%). Maximum likelihood phylogenetic analysis resolved two distinct clades with medium bootstrap support when naturalized populations were aligned in conjunction with reference native lineages. The haplotype network revealed a close association between naturalized populations and four main haplotypes representative of three native ecotypes or lineages from western North America (rainbow trout, steelhead trout and redband trout). These results indicate a genetic population structuring for naturalized rainbow trout from southern Chile and an origin probably represented by multiple lineages sources. Thus, mitochondrial DNA data strongly suggest that stocking of rainbow trout from different origins may have occurred during or after the initial introduction efforts.

## Introduction

Rainbow trout *Oncorhynchus mykiss* (Walbaum, 1792) is native to the Pacific basin, including the North American Pacific coast and the rivers that drain into it, from Alaska to the north of Mexico, as well as southwards on the Asian shore (MacCrimmon, 1971; Ade, 1989). Introduced to temperate freshwaters worldwide in the 1870s (MacCrimmon, 1971), a naturalized population of this species now exists in Central and South America, Australasia, Africa, Europe and the Indian subcontinent (Crawford and Muir, 2008). In fact, this species has been introduced into 99 countries, with populations being established in at least 53 of them (Gherardi, 2010). More recently, the global importance of farmed trout as a food source has likely expanded the distribution of rainbow trout as a consequence of escapes from fish farms (Stanković et al., 2015). The rainbow trout strain introduced outside of their native range comes from the McCloud River in northern California (MacCrimmon, 1971). Available data indicate that this stock was not initially pure because it probably was involved in mixing among the stream-resident form and the anadromous steelhead (Needham and Behnke, 1962). In addition, during the domestication process that takes place over several generations, the initial stock was also subjected to varying degrees of hybridization with other source populations (Busack and Gall, 1980; Gall and Crandell, 1992; Behnke, 2002). As a consequence, the taxonomic identity of this stock has not been elucidated to date.

Rainbow trout in their native range is a polytypic species that is characterized by striking phenotypic and genetic variation among populations. Thus, populations may differ in coloration and spotting, season of spawning, propensity for anadromy, morphology, and temperature and alkalinity tolerance (Schreck and Behnke, 1971; Gold, 1977; Behnke, 1992; Hershberger, 1992; Taylor et al., 2010). At the genetic level, this variability is reflected in great genetic divergence among populations (up to 75%) when they are classified according to natural diversity or distribution range, as has been reported in, for example, western North America (Utter et al., 1980; Nielsen et al., 1994; Nielsen et al., 1997; Bagley and Gall, 1998; Nielsen et al., 1998). In this region, 10 native groups have been recognized using morphological data, with some varieties named redband trout, steelhead trout, golden trout, freshwater resident and coastal steelhead trout (Behnke, 1992). These groups represent five groups of mitochondrial DNA (mtDNA) genotypes according to phylogenetic analysis, a result that mostly shows agreement with the morphological data (Bagley and Gall, 1998). This strong genetic structuring of rainbow trout has also been supported in other regions across their native distribution range (Nielsen et al., 1997; Narum et al., 2004; Brunelli et al., 2010; Taylor et al., 2010; Abadía-Cardoso et al., 2015).

Historical records indicate that rainbow trout were first introduced into southern Chile from Germany between 1905 and 1910 (Golusda, 1927; Hershberger, 1992). It is likely that the strain of this introduced species originates from California (Baird station breeding site on the McCloud River), since rainbow trout translocated from Germany to our country appears to have originated from this geographical area (MacCrimmon, 1971; Stanković et al., 2015). Young fish from these stocks were transferred to different central and southern rivers of the country (Golusda, 1927; Dazarola, 2019), where the establishment of self-sustaining populations occurs in various watersheds (Campos, 1970; Soto et al., 2006; Arismendi et al., 2014). In southern Chile, the first stocking efforts occurred in 1910, using fry obtained from eggs that had recently arrived from Germany and included the Maullín River, Petrohué River, Chamizas River, Coihuin River, Rahue River and Puelo River (Dazarola, 2019). Subsequently, and until 1930, fry obtained from breeders reared at the Río Blanco state fish farm were used to continue the introduction process in this geographic area. Similarly, from 1916 onwards another state center located further south, the Lautaro fish farm, also contributed to the process of introducing rainbow trout into the southern basins (Dazarola, 2019). Recently, several culture strains of rainbow trout imported from 1980 onwards from different countries for aquaculture purposes (Monzón-Argüello et al., 2014), have increased the diversity of trout in Chilean water bodies as a result of deliberate seeding or unintentional releases from farming centers, especially in lakes used for intensive fish farming activities (Arismendi et al., 2009; Arismendi et al., 2014). In fact, genetic studies suggest that part of this increased diversity has occurred through interbreeding with existing naturalized populations (Consuegra et al., 2011).

In Chile, there is little information on the origin or the lineages that compose the rainbow trout populations spread in natural environments and data on their evolutionary relationships with different varieties of North America. Genetic information available is limited to allozyme, microsatellite and single nucleotide polymorphisms (SNPs) variabilities in naturalized populations distributed in the south and north of the country (Gajardo et al., 1998; Consuegra et al., 2011; Benavente et al., 2015; Cárcamo et al., 2015; Canales-Aguirre et al., 2018). These studies helped to elucidate the diversity and structuring of rainbow trout inhabiting Chile, showing levels of genetic diversity similar to trout in its native distribution range (Gajardo et al., 1998; Consuegra et al., 2011). A marked genetic differentiation between populations located in the northern distribution of the country has also been recorded (Cárcamo et al., 2015).

mtDNA has many attributes that make it particularly suitable for population genetic analysis, including rapid rate of evolution, lack of recombination, and maternal inheritance (Liu and Cordes, 2004). Due to the rapid rate of evolution of mtDNA, the analysis of this molecule has proven useful in clarifying relationships among closely related species. Thus, mtDNA genealogies have been used extensively to trace processes at the population level and the phylogenetic diversification of taxa in relation to their geographical distribution (Avise et al., 1987). Among the different mtDNA markers available, the control region (CR) (or D-loop) has become an ideal marker for characterizing geographical patterns of genetic variation within and between populations since this marker contains many polymorphic sites (Palumbi, 1996). In the case of salmonids, this marker shows high performance for identifying the origins of introduced populations and for assessing genetic variation between wild and introduced populations (Bernatchez et al., 1992; Quinn et al., 1996; Burger et al., 2000; Colihueque, 2015). In addition, phylogenetic analysis based on CR marker may provide essential information on historical patterns of introduction and the concomitant evolutionary process that may underpin the naturalization process. In rainbow trout, there are only two studies using CR to analyze the origins of populations introduced outside their distribution ranges: one study was conducted in a river in Argentinian Patagonia (Riva Rossi et al., 2004) and the other identified the source of European populations (Stanković et al., 2016). However, no study has focused on self-sustaining populations from Chile. These studies support that introduced populations in these regions are derived from multiple sources, although parental populations mostly belong to rainbow trout from central and northern California.

In Chile, the efforts to stock rainbow trout strains in natural environments, as well as the policies supporting these activities, have mostly not been documented. Thus, unravelling the origin of naturalized populations is relevant to determine the existence of different lineages, to assess their distribution pattern across different hydrographic basins and to support the stocking efforts aimed at reinforcing valuable varieties, for example, for recreational fishing purposes. To assess the genetic structure and the origin of the naturalized population of rainbow trout distributed in southern watersheds of the country (39°–41° S latitude), we examined the CR sequence variations. With this aim, sequence data were compared with reference sequences from western North America rainbow trout strains recovered from public databases to calculate genetic divergence parameters and the phylogenetic relationships to help to clarify its probable origin. We hypothesized that introduced populations of rainbow trout in southern Chile are derived from multiple sources. This characteristic could be attributable to either the co-existence of different strains or the interbreeding of escaped cultured strains with existing naturalized populations.

## Materials and Methods

### Sampling and DNA Extraction

**Figure 1:**
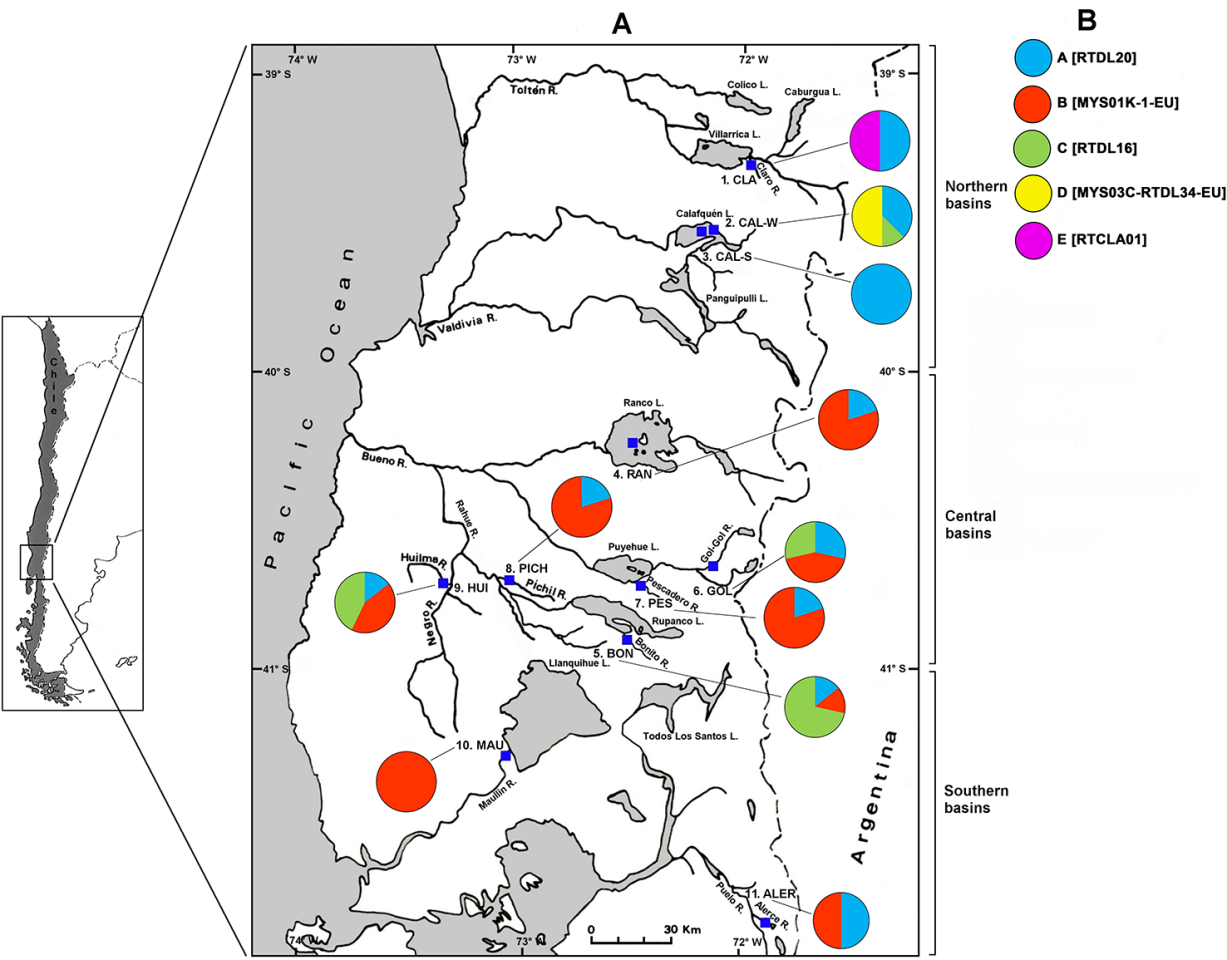
Map of collection sites and haplotype frequency of naturalized populations of rainbow trout from southern Chile. **(A)** Collection sites (blue squares) were as follows: 1) CLA (Claro River), 2) CAL-W (Calafquén Lake-winter spawn), 3) CAL-S (Calafquén Lake-spring spawn), 4) RAN (Ranco Lake), 5) BON (Bonito River), 6) GOL (Gol-Gol River), 7) PES (Pescadero River), 8) PICH (Pichil River), 9) HUI (Huilma River), 10) MAU (Maullín River), and 11) ALER (Alerce River). Populations were classified as belonging to different geographic groups (indicated in brackets at the righ side of the figure) as follows: northern basins group: 1 + 2+3 populations, central basins group: 4 + 5+6+7+8+9 populations and southern basins group: 10 + 11 populations. **(B)** Haplotype assignments scheme for the mtDNA control region based on reference haplotypes. Haplotype assignments are presented with specific colours within coloured pie charts that represent their relative frequency within each population. Haplotype labels correspond to those indicated in **Supplementary Table 1**.

Specimens of *O. mykiss* were collected in eleven localities from southern Chile (39°–41° S latitude) from 1999 to 2017 (**Table 1** and **Figure 1A**). Sampling localities were as follows: Claro River (CLA, n = 2), Ranco Lake (RAN, n = 5), Bonito River (BON, n = 7), Gol-Gol River (GOL, n = 7), Pescadero River (PES, n = 5), Huilma River (HUI, n = 7), Pichil River (PICH, n = 5), Maullin River (MAU, n = 6) and Alerce River (ALER, n = 8). In addition, two broodstocks collected from Calafquén Lake were included, which comprised winter-spawners (CAL-W, n = 8) and spring-spawners (CAL-S, n = 8), which were considered as distinct populations due to their different spawning time. These localities belong to five different river basins, which originate in the western Andean Mountains at an altitude above 1,000 m and flow in a relatively straight line until reaching the Pacific Ocean. All sampled fishes were captured by angling or electro-fishing. Fin clips from the dorsal fin of each specimen were obtained and immediately fixed in 80% ethanol until DNA was extracted. DNA was extracted using the phenol–chloroform method, as described in Taggart et al. (1992). Extracts were standardized at a concentration of 100 ng/µl in Tris-EDTA buffer pH 8.0.

**Table 1:** Sampling locations of naturalized rainbow trout specimens from southern Chile collected in this study.

Sampling Location	Sample Size	Code	Sampling Date	Geographic Coordinates	Province	Basin
1. Claro River	2	CLA	Oct-1999	39°17’27.0’’S 71°55’58.0’’W	Cautín	Toltén River basin
2. Calafquén Lake winter-spawners	8	CAL-W	Jul-2014	39°29’27.57’’S 72°09’44.42’’W	Cautín	Valdivia River basin
3. Calafquén Lake spring-spawners	8	CAL-S	Oct-2007	39°29’27.57’’S 72°09’44.42’’W	Cautín	Valdivia River basin
4. Ranco Lake	5	RAN	Feb-2017	40°17’02.0’’S 72°24’48.2’’W	Ranco	Bueno River basin
5. Bonito River	7	BON	Jul-2017	40°53’28.3’’S, 72°27’41.8’’W	Osorno	Bueno River basin
6. Gol-Gol River	7	GOL	Jun-2007	40°39’47.9’’S 72°15’00.4’’W	Osorno	Bueno River basin
7. Pescadero River	5	PES	Oct-2007	40°42’54.7’’S 72°24’16.2’’W	Osorno	Bueno River basin
8. Pichil River	5	PICH	Dec-1999	40°43’07.0’’S 72°57’01.0’’W	Osorno	Bueno River basin
9. Huilma River	7	HUI	Jan-2000	40°43’05.0’’S 73°13’16.0’’W	Osorno	Bueno River basin
10. Maullin River	6	MAU	Jun-2016	41°16’06.2’’S 73°00’34.9’’W	Llanquihue	Maullin River basin
11. Alerce River	8	ALER	Aug-2017	41°52’17.0’’S, 71°55’13.4’’W	Llanquihue	Puelo River basin

### PCR and Sequencing

The control region sequences were amplified using the primer pair of Ctrl Reg-L19 (5’-CCACTAGCTCCCAAAGCTA-3’) and Ctrl Reg-03-R (5’-GTGGGTAACGGGCAATAAGA-3’). PCR amplification was carried out in 45 µl using a reaction mix composed of 9 µl Taq polymerase buffer A (1×), 0.9 µl of dNTPs (0.2 mM), 1.35 µl of MgCl_2_ (1.5 mM), 0.9 µl of each primer (0.2 µM), 0.18 µl of Taq DNA polymerase (0.02 U/µl) (Kapa Biosystems), 9.0 µl of template DNA (20 ng/µl), and 22.77 µl of DNAse-free and RNAse-free distilled water (Gibco). The thermal profile was performed as follows: initial denaturation at 94°C for 2 min followed by 25 cycles at 94°C for 45 s, 62°C for 45 s with −0.5°C per cycle and 72°C for 55 s, then 15 cycles at 94°C for 45 s, 54°C for 45 s, 72°C for 55 s and a final extension step at 72°C for 5 min. PCR products were visualized on 2% agarose gels, and prior to sequencing, these products were cleaned with a QIA-quick Gel Extraction Kit (Qiagen). PCR products were bi-directionally sequenced on an Applied Biosystems ABI377 automated sequencer. The sequences from forward and reverse reads were aligned and edited using GENEIOUS 4.0.2 software (Biomatters Ltd.) to obtain consensus sequences for all individuals. The sequences were deposited in GenBank under accession numbers MN166836–MN166903.

### Population Genetic Structure

Genetic variation within populations was assessed by the number of haplotypes, haplotype diversity, nucleotide diversity, polymorphic sites and average number of pairwise nucleotide differences using DnaSP 5.1 software (Librado and Rozas, 2009). Coalescent analysis based on neutral infinite-sites model and assuming a large constant population size (Hudson, 1990), with theta per gene being estimated from the data, no recombination and 1,000 replicates settings, was also performed using the same software. This approach allowed us to estimate the average number and variance (95% confidence interval) of expected haplotypes to assess whether sampling effort was sufficient to capture the genetic diversity. Global *F* *_ST_* was calculated according to Hudson et al. (1992). Pairwise population structure was evaluated a posteriori by means of *F* *_ST_*; and significance was tested using 10,000 permutations with a level of significance of α = 0.05. The exact test of population differentiation (Raymond and Rousset, 1995) based on haplotype frequency was also applied as implemented using the Markov chain with 10,000 steps. Analysis of molecular variance (AMOVA) (Excoffier et al., 1992) implemented in the program Arlequin ver 3.5 (Excoffier and Lischer, 2010) was used to assess the geographical pattern of population subdivision. To carry out this analysis, populations were separated into three groups according to the latitudinal location of the basins: 1) northern basins (N-basins, 39°–40° S), 2) central basins (C-basins, 40°–41° S) and 3) southern basins (S-basins, 41°–42° S) (**Figure 1A**). This division considered the possible existence of differences in the haplotype frequencies among groups due to the differential effect of the unintentional escape of cultured strains from trout farming centers, especially in C-basins (Arismendi et al., 2009; Arismendi et al., 2014). In AMOVA the correlation of haplotype frequency was used as an F-statistic analogue at various hierarchical levels. The *F* *_ST_* estimates the proportion of genetic variation within populations relative to the genetic variation from the whole sample, whereas *F* *_CT_* estimates the proportion of genetic variation among groups of populations relative to the whole sample, and *F* *_SC_* estimates the variation among populations relative to a grouping of populations. The significance of these F-statistic analogues was evaluated by random permutations of sequences among populations.

### Phylogenetic Reconstruction and Genetic Distances

Phylogenetic relationships for haplotypes were reconstructed using maximum likelihood (ML) by heuristic search methods in MEGA 5.05 software (Tamura et al., 2011). The corresponding sequence of *Oncorhynchus clarki* (accession no AF044167) was used as an outgroup for rooting purposes. The best-fit nucleotide substitution model was determined using jModelTest 2.1 (Darriba et al., 2012) based on the Bayesian Information Criterion (BIC). The best model was then used with ML analysis to construct a ML tree. The best fit-model of nucleotide substitution was Hasegawa-Kishino-Yano, with a proportion of invariable sites (HKY+I) and parameter estimates for base frequencies of A = 0.3280, T = 0.3188, C = 0.2039 and G = 0.1493, and proportion of invariable sites of 0.9120. The consistency of topologies (nodal support) was estimated using a bootstrap approach with 1,000 bootstrap replications (Felsenstein, 1985). Rainbow trout CR sequences from southern Chile were aligned against CR reference sequences (left domain) previously published by Bagley and Gall (1998) to Nevada, Idaho and Northern California populations, which represent the natural diversity of 10 native groups of *O. mykiss* according to Behnke (1992) (**Supplementary Table 1**). These groups represent different ecotypes of rainbow trout, and possibly subspecies-level categories, for example, McCloud River redband (*O. mykiss stonei*), interior redband (*O.m. gairdneri*), Volcano Creek golden trout (*O.m. aguabonita*), Kern River rainbow trout (*O.m. gilberti*) and coastal steelhead (*O.m. irideus*). The CR reference sequences involved twelve haplotypes and most present high frequency in native populations. These haplotypes are representative of the five mtDNA haplogroups reported by Bagley and Gall (1998) (haplogroups I, II, III, IV and V) and using this means of selection (1‒5 haplotype per haplogroup), it was possible to examine what part of the genetic variation of native populations was contained in Chilean populations. An interesting haplotype within this dataset is the RTDL26 (haplogroup I), which has a high frequency (100%) in the McCloud River from northern California. This drew our attention because historical records identify this site as the place where the rainbow trout strain introduced outside its native range originated from. The McCloud River includes populations of the redband ecotype (Bagley and Gall, 1998). We also included two additional haplotypes published by Stanković et al. (2016), named MYS01K-1-EU and MYS03C-RTDL34-EU. The MYS01K-1-EU haplotype has a high frequency in Pacific Northwest populations from North America (25–100%). Interestingly, this haplotype has also been registered in Europe, but at a low frequency. For example, in genuine self-sustaining populations of this continent this haplotype shows a frequency of only 7.5%. The MYS03C-RTDL34-EU haplotype has been reported at low frequency in Europe, but it is more frequent in native populations, particularly those from Oregon (Brunelli et al., 2010). Pairwise genetic distances among naturalized populations of rainbow trout from southern Chile and native populations were also estimated by using p-distance that was calculated using MEGA v. 5.05 software. This index estimates the proportion of nucleotide sites at which the two sequences to be compared are different. Relationships among CR haplotypes were also reconstructed based on the median joining network (MJN) implemented in Network program ver. 5.0.0.3 (Bandelt et al., 1999). In this analysis, the data set of native populations was classified by ecotypes. As our data and those from Bagley and Gall (1998) used different sampling sizes, nodes of MJN were represented by relative frequencies.

## Results

### Sequence Variability

A 435-bp fragment of the mtDNA control region sequences was obtained from 68 samples. Five variable sites were identified (1.15%), including four parsimony informative sites and one singleton variable site. The nucleotide composition was A, 32.5%, C, 20.8%, G, 15.2%, and T, 31.5%. The A–T content (64.0%) was higher than the G–C content (40.0%), showing an overall transition/transversion ratio of 2.94. Five haplotypes were defined (**Table 2**), with two haplotypes representing approximately 76% of the individuals sampled (Haplotype A, 33.8%; Haplotype B, 42.6%), while the remaining haplotypes (Haplotypes C, D and E) were sampled below 16.2%. The sequence shows an overall haplotype diversity (*H*) of 0.684 ± 0.030 and a nucleotide diversity (*Π*) of 0.00460 ± 0.00012 (**Table 3**). The coalescent analysis indicated that populations approached the estimated total allele number relatively well, since most populations show slight negative deviations with respect to the average number of expected haplotypes, although all fell within a 95% confidence interval. Thus, this analysis indicated that possibly most alleles were captured. The highest level of *Π* was found in CLA (0.01149), while the lowest value was found in BON (0.00263) and in sampling localities with only one haplotype. The highest level of *H* was found in CLA (1.000), while RAN, PES and PICH had the lowest *H* (0.400), not considering populations with only one identified haplotype (i.e., MAU). The average number of pairwise nucleotide differences was *k* = 1.999, and ranged from *k* = 0.0000 in CAL-S and MAU to *k* = 5.0000 in CLA.

**Table 2:** Aligment of mtDNA control region haplotypes of variable positions and distribution of mtDNA control region haplotypes in sampling localities.

	Variable Sites					Localities												
Haplotype	3	92	244	245	325	ALER	BON	CAL-W	CAL-S	CLA	GOL	HUI	RAN	MAU	PES	PICH	Total	%
A [RTDL20]	A	T	A	T	A	4	1	3	8	1	2	1	1	0	1	1	23	33.8
B [MYS01K-1-EU]	.	A	G	C	G	4	1	0	0	0	3	3	4	6	4	4	29	42.6
C [RTDL16]	.	.	.	.	G	0	5	1	0	0	2	3	0	0	0	0	11	16.2
D [MYS03C-RTDL34-EU]	.	A	.	C	G	0	0	4	0	0	0	0	0	0	0	0	4	5.9
E [RTCLA01]	G	A	G	C	G	0	0	0	0	1	0	0	0	0	0	0	1	1.5

In brackets are indicated the haplotype codes that correspond to the reference haplotypes described for North American populations (see **Supplementary Table 1**), except those for haplotype E that was reported in the current study. Locality names are similar to those in **Table 1.**

**Table 3:** Summary of genetic diversity indices for eleven naturalized populations of rainbow trout based on mtDNA control region.

Populations	*n*	*h*	*s*	*H*	*Π*	*k*	*Neh*
ALER	8	2	4	0.571 ± 0.094	0.00525 ± 0.00087	2.286	3.798 (2.000–6.000)
BON	7	3	4	0.524 ± 0.209	0.00263 ± 0.00136	1.143	2.804 (1.000–5.000)
CAL-W	8	3	3	0.679 ± 0.122	0.00386 ± 0.00061	1.679	3.408 (1.000–6.000)
CAL-S	8	1	0	0.000 ± 0.000	0.00000 ± 0.00000	0.000	
CLA	2	2	5	1.000 ± 0.500	0.01149 ± 0.00575	5.000	1.838 (1.000–2.000)
GOL	7	3	4	0.762 ± 0.115	0.00504 ± 0.00089	2.190	3.569 (1.000–6.000)
HUI	7	3	4	0.714 ± 0.127	0.00460 ± 0.00089	2.000	3.400 (1.000–6.000)
RAN	5	2	4	0.400 ± 0.237	0.00368 ± 0.00218	1.600	2.694 (1.000–5.000)
MAU	6	1	0	0.000 ± 0.000	0.00000 ± 0.00000	0.000	
PES	5	2	4	0.400 ± 0.237	0.00368 ± 0.00218	1.600	2.694 (1.000–4.000)
PICH	5	2	4	0.400 ± 0.237	0.00368 ± 0.00218	1.600	2.706 (1.000–5.000)
Total	68	5	5	0.684 ± 0.030	0.00460 ± 0.00012	1.999	7.597 (4.000–12.000)

n, sample sizes; h, number of haplotypes; s, number of polymorphic sites; H, haplotype diversity ± standard deviation; Π., nucleotide diversity ± standard deviation; k, average number of pairwise nucleotide differences; Neh, average number of expected haplotypes with 95% confidence interval in parenthesis based on coalescent simulations.

### Population Genetic Structure

Global *F* *_ST_* was 0.169, indicating moderate genetic differentiation among populations. Pairwise *F* *_ST_* estimates indicated that within basin groups (N-, C- and S-basins) most differences among populations were not significant (*P* >0.05). However, among basin groups, especially between the N- and C- or S-basins, several populations showed significant differences (*P* <0.05) (**Table 4**). The exact test of population differentiation corroborated the difference recorded among these groups (6,000 Markov steps, *P* <0.001). AMOVA based on haplotype frequency (**Table 5**) revealed that most variations were explained mainly by differences within populations (61.65%) (*F* *_ST_* = 0.38347, P <0.05) and among groups (20.82%) (*F* *_CT_* = 0.20818, *P* <0.05). Only 17.53% of the variance was attributed to differences among populations within groups (*F* *_SC_* = 0.22137), a result that was in accordance with the pairwise *F* *_ST_* estimate.

**Table 4:** Pairwise *F* *_ST_* values in eleven naturalized populations of rainbow trout based on mtDNA control region.

Basin Location	Population	N-basin			C-basin						S-basin	
		CAL-W	CAL-S	CLA	GOL	HUI	RAN	PES	PICH	BON	MAU	ALER
N-basin^a^	CAL-W	0.00000										
	CAL-S	0.45714*	0.00000									
	CLA	0.05398	0.62791	0.00000								
C-basin^b^	GOL	0.16111	0.49206*	0.03020	0.00000							
	HUI	0.22064*	0.60591*	0.14662	−0.13021	0.00000						
	RAN	0.39336*	0.80907*	0.37198	0.01228	0.09468	0.00000					
	PES	0.39336*	0.80907*	0.37198	0.01228	0.09468	−0.25000	0.00000				
	PICH	0.39336*	0.80907*	0.37198	0.01228	0.09468	−0.25000	−0.25000	0.00000			
	BON	0.29534*	0.71289*	0.30835	0.07353	−0.01111	0.45209*	0.45209*	0.45209*	0.00000		
S-basin^c^	MAU	0.61984*	1.00000*	0.80645*	0.30290	0.34477	0.04000	0.04000	0.04000	0.67315*	0.00000	
	ALER	0.23077	0.42857	0.08046	−0.03448	0.10251	0.01408	0.01408	0.01408	0.36000*	0.37662	0.00000

*P <0.05 (10,100 permutations). ^a^northern basins, ^b^central basins, ^c^southern basins.

**Table 5:** Analysis of molecular variance (AMOVA) among eleven samples of rainbow trout separated in three groups (Northern, Central and Southern basins) using sequences of the mtDNA control region.

Source of Variation	df	Sum of Squares	Covariance Components	Percentage Variation	Fixation Indices	Probability
Among groups	2	4.638	0.07864 Va	20.82	*F* *_CT_* = 0.20818	0.01564*
Among populations within groups	8	4.999	0.06621 Vb	17.53	*F* *_SC_* = 0.22137	0.00293*
Within populations	57	13.275	0.23289 Vc	61.65	*F* *_ST_* = 0.38347	0.00000*
Total	67	22.912	0.37775			

* P < 0.05 (10,000 permutations).

### Phylogenetic Analysis and Genetic Distances

**Figure 2:**
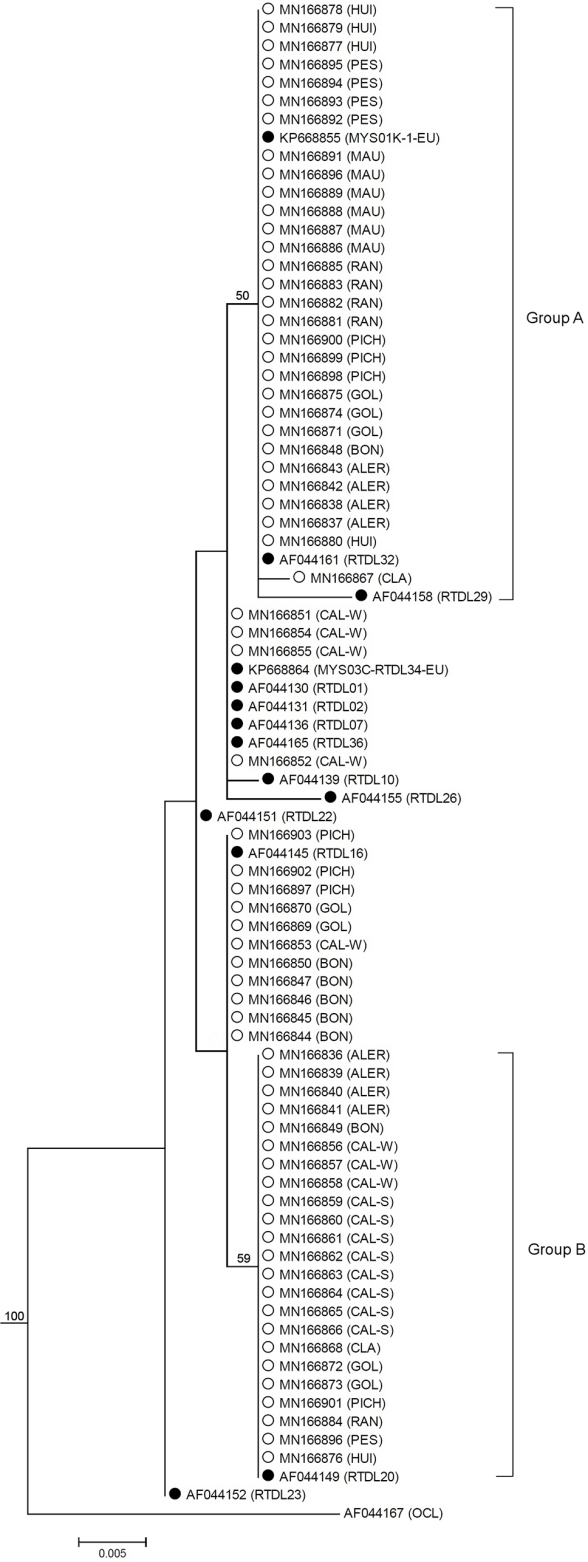
Maximum likelihood consensus tree for Chilean naturalized rainbow trout populations based on the mtDNA control region. Samples analyzed in this study (white circles) are indicated by the Genbank accession numbers and population code are in parenthesis. Reference haplotypes (black circles) are indicated by the Genbank accession numbers, while the haplotype labels for native ecotypes are in parenthesis. Haplotype labels for native ecotypes correspond to those indicated in **Supplementary Table 1**. The values on the nodes indicate the bootstrap support for each node; values >50% are shown. The branch lengths are drawn proportional to the relative amount of evolutionary change. Scale indicates the sequence divergence estimated from the number of nucleotide substitutions per site.

**Figure 3:**
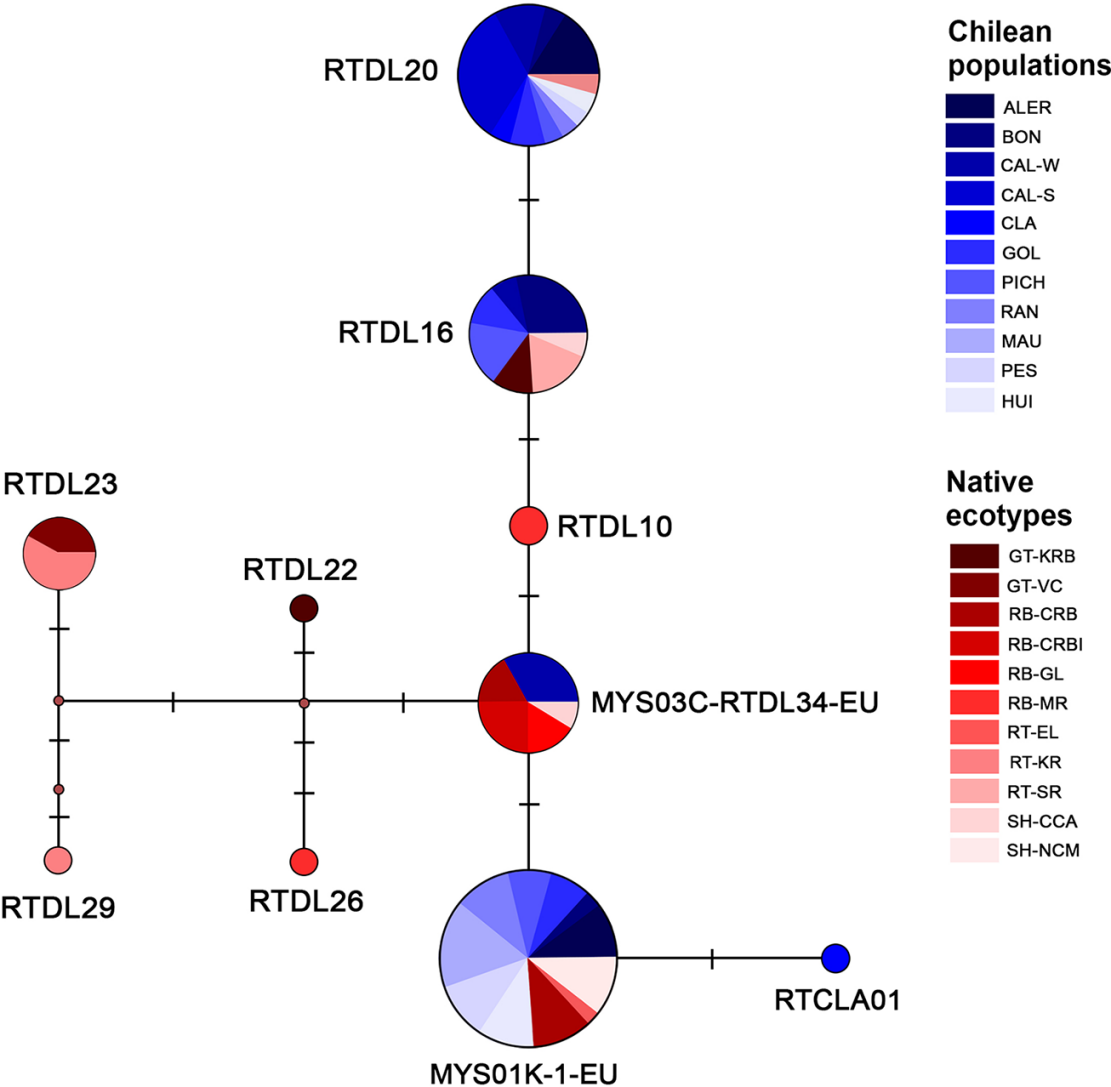
Haplotype network of Chilean naturalized rainbow trout populations in conjunction with native ecotypes based on the mtDNA control region. The area of each circle is proportional to the number of individuals. Each perpendicular line between haplotypes indicates a single mutational step. Labels for haplotypes and native ecotypes correspond to those indicated in **Supplementary Table 1**. RTCLA01 haplotype corresponds to a new haplotype found in this study.

The multiple sequence alignment (435 align sites) of the 68 sequences from naturalized rainbow trout populations from southern Chile in conjunction with 14 reference haplotypes of native populations, revealed the existence of five different haplotypes in Chilean population (A, B, C, D and E) (**Table 2**). The haplotypes A, B, C and D corresponded to the reference haplotypes RTDL20, MYS01K-1-EU, RTDL16, MYS03C-RTDL34-EU, respectively. In turn, haplotype E corresponded to a new haplotype called RTCLA01, which was recorded only in the CLA population. The haplotypes RTDL20 and RTDL16 belong to haplogroup II of Bagley and Gall (1998). In addition, the RTDL26 haplotype that is characteristic of the source populations of the McCloud River was not found in any population analyzed. Phylogenetic analysis showed most naturalized populations of rainbow trout from southern Chile clustered into two groups of different sizes (labelled Group A and Group B) but with moderate consistency support (**Figure 2**). Group A clustered naturalized populations with two reference haplotypes: MYS01K-1-EU, a haplotype that shows a high frequency in the steelhead trout from north of Cape Mendocino and redband trout from the Columbia River basin lineages of *O. mykiss* of the Pacific Northwest from North America (i.e., British Columbia, Washington and Oregon); and haplotype RTDL32 found at low frequency in a rainbow trout lineage of the Eagle Lake from California (**Supplementary Table 1**). Group B included the reference haplotype RTDL20, which has been found at low frequency in a hatchery stock consisting of a lineage from southern California, represented by the Kern River basin rainbow trout. Moreover, in both groups a mixing of Chilean populations of *O. mykiss* from different basins was observed. This pattern was also recorded at population level, since samples from some populations (i.e., HUI, PES, RAN, PICH, GOL and ALER) clustered into the two different groupings in the haplotype tree. The haplotype network revealed four main haplotypes, RTDL20, RTDL16, MYS03C-RTDL34-EU and MYS01K-1-EU, separated by one or two mutations (**Figure 3**). These haplotypes shared individuals from both Chilean populations and native ecotypes, with most Chilean populations being grouped into RTDL20, RTDL16 and MYS01K-1-EU haplotypes. Moreover, our results also suggest that the haplotype distribution appeared to have a geographic structure, since RTDL20 was more frequent in populations from N-basins, in contrast to MYS01K-1-EU that was only recorded in populations from C- and S-basins (**Figure 1**). The comparison of rainbow trout populations from southern Chile with native populations classified by ecotypes revealed lower mean genetic p-distance with steelhead and redband trout than with golden and rainbow trout (0.42–0.46% and 0.55–0.59%, respectively) (**Table 6**). This result was corroborated at the population level because most populations from southern Chile presented a p-distance lower than the overall mean p-distance (0.48%) in redband (7/11) and steelhead ecotypes (8/11) than in golden (3/11) and rainbow trout (1/11) ecotypes.

**Table 6:** Genetic distances based on p-distance between Chilean naturalized populations and native populations of rainbow trout classified in four major ecotypes using sequences of the mtDNA control region.

Populations	Golden Trout (n = 6)	Redband Trout (n = 14)	Rainbow Trout (n = 10)	Steelhead (n = 6)
ALER	0.57 ± 0.24	0.50 ± 0.23	0.61 ± 0.25	**0.46 ± 0.22**
BON	**0.36 ± 0.18**	0.52 ± 0.27	**0.45 ± 0.20**	0.51 ± 0.28
CAL-W	**0.43 ± 0.20**	**0.42 ± 0.19**	0.52 ± 0.22	**0.47 ± 0.23**
CAL-S	0.50 ± 0.29	0.76 ± 0.37	0.57 ± 0.27	0.77 ± 0.37
CLA	0.69 ± 0.26	0.62 ± 0.25	0.72 ± 0.27	0.57 ± 0.24
GOL	0.50 ± 0.22	**0.47 ± 0.22**	0.55 ± 0.23	**0.44 ± 0.22**
HUI	**0.47 ± 0.21**	**0.44 ± 0.22**	0.53 ± 0.22	**0.41 ± 0.21**
RAN	0.62 ± 0.30	**0.35 ± 0.17**	0.63 ± 0.28	**0.28 ± 0.14**
MAU	0.65 ± 0.35	**0.25 ± 0.17**	0.64 ± 0.31	**0.15 ± 0.09**
PES	0.62 ± 0.30	**0.35 ± 0.17**	0.63 ± 0.28	**0.28 ± 0.14**
PICH	0.62 ± 0.30	**0.35 ± 0.17**	0.63 ± 0.28	**0.28 ± 0.14**
Mean	0.55 ± 0.26	0.46 ± 0.22	0.59 ± 0.26	0.42 ± 0.26

Bold number indicates a p-distance below the overall mean p-distance (p-distance = 0.48%).

## Discussion

### Genetic Structure of Naturalized Rainbow Trout Populations

Our results based on the mtDNA CR marker, which involved the analysis of eleven populations belonging to five basins from southern Chile, indicate moderate but significant genetic differentiation among populations. The AMOVA results also support that this genetic variation originates primarily from differences within populations. However, among group differences were also significant, i.e. differences between northern and central or southern basins, which suggests that genetic divergence has a geographic structure. Considering that sample sizes per population were limited in some cases (e.g., Claro River), estimation of genetic variation among populations should be considered with caution. Nevertheless, this result suggests the occurrence of a substantial degree of differentiation of populations among different basins, which concurs with the possible multiple origin hypothesis of naturalized rainbow trout populations from southern Chile.

The level of genetic structuring recorded in our study (*F* *_ST_* = 0.169) is higher than that reported in other studies of naturalized populations of rainbow trout distributed in southern Chile. Since in previous studies the genetic analysis was usually limited to one or few basins located within a more restricted geographic area (Gajardo et al., 1998; Consuegra et al., 2011; Canales-Aguirre et al., 2018), the high level of genetic structuring registered in our study may be related to the analysis of a broader geographic scale. In this regard, different factors, such as isolation by distance, among others, may play a significant role in increasing the differentiation among basins. In contrast, Gajardo et al. (1998) using allozymic markers, reported low genetic structuring in three naturalized populations of rainbow trout (*F* *_ST_* = 0.052) collected in tributaries at two Andean lakes (Puyehue and Rupanco lakes), both located in the same river basin (Bueno River basin). Consuegra et al. (2011), using microsatellite markers, also found a low level of genetic structuring (*F* *_ST_* = 0.073) in fifteen naturalized populations of this species that inhabit a relatively restricted geographic area from the mainland and Chiloé Island of the Región de Los Lagos. In fact, no significant isolation by distance was detected for these populations. In addition, Canales-Aguirre et al. (2018) using SNP markers, found higher genetic divergence than previous studies among naturalized populations belonging to the same basin that inhabit different inlets from Llanquihue Lake and from Todos Los Santos Lake (pairwise *F* *_ST_* = 0.102–0.156). It should be noted that since the mtDNA Control Region is a more variable genetic marker than those used in the aforementioned reports, due to its higher mutation rate and smaller effective population size (Liu and Cordes, 2004), the high level of genetic structuring reported in our study may be attributable in part to this factor. However, if we compare our data with those of introduced populations from Europe that were recorded using the same genetic marker, the level of genetic structure is about half of that reported in these populations (*F* *_ST_* = 0.169 vs. 0.383), which reveals that the populations studied present a relatively moderate level of structuring.

The existence of a strong genetic differentiation within populations in our study could be interpreted as the occurrence of mixing of individuals from different sources or genetic origins within populations. In fact, the level of overall haplotype diversity found in our study (0.684) was in the upper limit of that reported in native populations from western North America (0.352–0.825) (Stanković et al., 2016), which provides further support for the possible occurrence of this genetic pattern in naturalized rainbow trout from Southern Chile. The rainbow trout has been introduced in several countries around the world, with well-established populations being reported in many of them (Gherardi, 2010). However, to date, the genetic composition of most of these populations has not been determined. Some studies, such as those investigating European populations (Stanković et al., 2016), suggest that in this continent, the naturalized population of rainbow trout has a multiple origin. This statement was based on the presence of multiple native lineages and high levels of allelic richness and genetic diversity, including the occurrence of some degree of geographic break of the haplotypes across its distribution range. Our results agree with this result, revealing that rainbow trout may have experienced a complex introduction process, at least in some regions.

It should be noted, however, that genetic differentiation among populations may also arise through genetic drift at the time of colonization by founder effects, or even after that time, since this process may promote the allele fixation in populations, especially in those that present small effective sizes. In other introduced salmonid, such as the chinook salmon, *Oncorhynchus tshawytscha*, that has been transplanted from California to New Zealand, this process seems to have played an important role in shaping the genetic differentiation among populations (Quinn et al., 1996). For example, in this species the allozyme and mtDNA evidence suggests the occurrence of a marked genetic drift as a consequence of a bottleneck that was followed by a larger and more stable population size. This process would explain the particular clustering of the introduced populations of Chinook salmon compared with those that inhabit the native range in the Sacramento River. In the case of the naturalized populations of rainbow trout studied here, the occurrence of the genetic drift cannot be ruled out. This is because available studies on some populations of the Llanquihue Lake that inhabit the inlet streams of this site indicate that the estimation of the annual number of breeders was usually low, which suggested that recently founded populations could be experiencing substantial genetic drift (Benavente et al., 2015).

### Origin and Diversity of Naturalized Rainbow Trout in Chile

The control region has been probed as an ideal marker for characterizing genetic variation and the origin of introduced populations of different salmonid species (Bernatchez et al., 1992; Quinn et al., 1996; Burger et al., 2000). In this study, we used this marker to trace the origin and genetic composition of the naturalized rainbow trout from southern Chile based on comparisons with the haplotypic data available for native populations from North America. We hypothesized that introduced populations of rainbow trout in Chile are derived from multiple sources due to historical and recent introduction records suggesting that different strains may compose the genetic pool of this salmonid. Our results support this hypothesis due to the following evidence: 1) the significant genetic differentiation recorded among populations, especially among those belonging to different river basins, 2) the high level of overall haplotype diversity registered in these populations in comparison with that reported in different native populations from western North America (Stanković et al., 2016), and 3) haplotype mixing in most populations, with some exhibiting up to three haplotypes (e.g. Bonito River, Gol-Gol River), including the clustering of some samples from certain populations into the two different groupings in the haplotype tree. The multiple origins hypothesis has been previously tested for naturalized populations of other geographic regions, such as Europe (Stanković et al., 2016), Argentina (Riva Rossi et al., 2004) and Missouri (Dillman and Koppelman, 2006). For example, Stanković et al. (2016) found that translocated populations from Europe present a higher level of allelic richness and genetic diversity than native populations and they are characterized by clustering in four well defined haplogroups. These data led them to conclude that the genetic pool of these populations should reflect a multiple origin. In addition, Riva Rossi et al. (2004) found a similar genetic pattern for naturalized anadromous and resident rainbow trout inhabiting a Patagonian river in Argentina (Santa Cruz River), although they demonstrated that most populations are likely to originate from a restricted region in North America, particularly the McCloud River in California. Similarly, Dillman and Koppelman (2006) found significant differences in mtDNA genotypes using cytochrome *b* gene among several naturalized and hatchery populations from Missouri, supporting that multiple sources of rainbow trout were probably represented. Taken together, these data indicate that naturalized rainbow trout populations in some regions around the world exhibit genetic heterogeneity.

Clarifying the mixing process of the Chilean naturalized population of rainbow trout may be a complex task. This complexity is encountered because the admixture process may have emerged by multiple independent introductions from divergent sources or by introductions of a mixed source. Historical records suggest that the latter alternative cannot be totally ruled out due to rainbow trout introduced outside of their native range comes from a stock of the McCloud River in northern California (MacCrimmon, 1971), whose genetic composition probably involved mixing among the stream-resident form and the anadromous steelhead (Needham and Behnke, 1962). However, contrary to our expectations, the origin of naturalized rainbow trout populations in southern Chile does not appear to have originated in the McCloud River, since the RTDL26 haplotype that is typical of this population was not found in any population analyzed. Thus, it is likely that other locations in northern California and southern Oregon could have contributed fish to early transplants, as has been stated by Behnke, (2002). For Chile, the historical record clearly indicates that rainbow trout was first introduced from Germany between 1905 and 1910 (Golusda, 1927), but after that time, there are no reliable historical records that enable us to determine the origin of the strains used for stocking in southern Chile. This situation is more complex because only after 2009 did the Chilean government apply regulations for stocking with naturalized species in Chile (Decreto Supremo 210, 2009), therefore, before this date, translocation of different strains within and between basins due to deliberate or unintentional seeding is likely to occur.

Recent data suggest that the independent introduction process may have played a role in southern Chile by means of escape events of different cultured strains from farming centers (Arismendi et al., 2009; Arismendi et al., 2014). Based on the present data we cannot determine what factors may be involved in the origin of the divergence pattern recorded in the studied populations, since this requires specific experimental designs (for example, by means of the comparison between hatchery strains of farming centers and naturalized populations), to ascertain the possible occurrence of recent admixture among both populations. However, it should be noted that this process is not unlikely to have occurred in these populations, because the central basins are a geographic area with intense trout farming activity in southern Chile due to the existence of several freshwater farming centers (Arismendi et al., 2014; Proyecto FIPA 2016-19, 2017). In fact, the smolt production of rainbow trout in net-pen installation of different lakes of this area (i.e., Llanquihue, Puyehue, Ranco and Rupanco lakes) varied, approximately, from one to 41 million during the period 1995 to 2005 (Arismendi et al., 2009). More importantly, it is assumed that of this total about 3‒5% may have constituted escaped fishes. This process is likely to have occurred in southern Chile basins where escaped individuals may interbreed with existing naturalized populations, which could modify the genetic composition by introgression, as indicated in recent studies (Consuegra et al., 2011). In the central basins this effect is expected to be more marked than in northern basins because there are about two fold more freshwater farming centers operating in the former than in the latter (Proyecto FIPA 2016-19, 2017). Moreover, in northern basins the trout farming activity has been more restricted due to legal constrains applied from 2001 onwards, especially in the lakes of this zone (Decreto Supremo 371, 2001). In the case of the southern basins, introduced populations are likely to be less affected by fish farming activity, since there are only few centers operating in this area, especially in Cochamo district where is located the Puelo River basin (only 3 of the 60 recorded for Región de Los Lagos) (Proyecto FIPA 2016-19, 2017

The possible admixture process is consistent with available studies performed in some sites of central basins, which suggest the occurrence of interbreeding of escapee individuals with those of existing naturalized populations Consuegra et al. (2011). For example, Benavente et al. (2015) provided evidence of the occurrence of this process, since a population of rainbow trout that inhabit a stream of Llanquihue Lake could have originated through the establishment of escaped farm broodstocks. Although specific experimental designs are required to address this question, some reports suggest that part of the genetic diversity currently found in naturalized populations from southern Chile has been shaped through the interbreeding of cultured strains with existing naturalized populations (Consuegra et al., 2011; Canales-Aguirre et al., 2018). Our result would also reflect this process, especially in sites where intensive trout farming activities related to smolt production have taken place in recent decades, as had occurred in Ranco Lake, Puyehue Lake and Llanquihue Lake (Arismendi et al., 2009). In these sites, the MYS01K-1-EU haplotype was dominant, in contrast with more northern populations (i.e., Calafquén Lake and Claro River), where trout farming activities for smolt production were excluded some time ago by the Chilean government (Decreto Supremo 371, 2001), and notably, this haplotype was absent. Thus, this pattern of haplotype distribution is likely to be related, at least in part, to the continuous impact of trout farming activities in these sites.

Another interesting issue addressed by our analysis was clarification of the lineage composition of naturalized populations of rainbow trout from southern Chile based on comparison with haplotypic data of native source populations, whose frequency has been associated with particular ecotypes (Bagley and Gall, 1998). As revealed by the haplotype network, most Chilean populations contained RTDL20, RTDL16 and MYS01K-1-EU haplotypes. Since these haplotypes are representative of specific native ecotypes, for example, RTDL20 is typical of the Kern River rainbow trout from California, RTDL16 is dominant in the coastal rainbow trout from the Sacramento River basin and MYS01K-1-EU is highly represented in the Steelhead trout from north of Cape Mendocino and redband trout from the Columbia River basin (see **Supplementary Table 2**), this result suggests that Chilean populations may be derived at least from three major ecotypes, namely, rainbow trout, steelhead trout and redband trout. Genetic distance analysis among Chilean and native populations classified by ecotypes corroborated in part this pattern, as Chilean populations were closely-related to steelhead trout and redband trout. However, it should be noted that other possible source populations cannot be ruled out, since the RTDL16 haplotype can also be found in steelhead trout from central California and the Golden trout of Kern River Basin, where it exhibits a relatively low frequency. Although the introduction process of the rainbow trout in certain regions around the world may have historical particularities, our result has good concordance with other reports. For example, in Europe, the haplotype data indicate that the great majority of parental populations of genuine self-sustaining populations are likely to have originated from golden trout (Kern River basin) and coastal rainbow trout (Sacramento River basin), and from steelhead (North of Cape Mendocino) and redband trout (Columbia River basin) (Stanković et al., 2016), since in these populations the RTDL16 and MYS01K-1-EU haplotypes were dominant, respectively. However, since our result indicates that the redband trout lineage is likely to be present in Chilean populations, these data suggest that the introduction process of rainbow trout in southern Chile has certain particularities in comparison with other regions. This idea is reinforced if we consider the findings of Riva Rossi et al. (2004), who found in a Patagonian river in Argentina a more restricted origin of naturalized populations, particularly from the McCloud River in California. In our case, this strain represented by the RTDL26 haplotype was not recorded in our study.

Moreover, since we found some haplotypes privative to certain basins, such as the MYS01K-1-EU haplotype that was only present in central and southern basins, these results suggest the possible existence of a geographic break of haplotypes for naturalized rainbow trout from southern Chile. Although further analyses of new populations are required to confirm this geographic structure, these results agree with other reports indicating that the rainbow trout may experience clinal breaks in the distribution of mtDNA haplotypes, either in native populations from western North America or in introduced population from Europe (Stanković et al., 2016). For example, in western North America the MYS01K-1-EU and the RTDL20 haplotypes are distributed mostly in the Northern and Southern part of this geographic area, respectively. Other studies that used the Y marker also revealed the occurrence of a geographic division of haplotypes of rainbow trout from western North America, but between the inland and coastal subspecies (Brunelli et al., 2010). Available studies show that the geographical break in the mtDNA haplotype distribution is not a rare phenomenon in fishes whose origin has been related to different factors. For example, an ancient allopatric divergence within separated watershed, the historical isolation of populations in different glacial refugia and the effect of geographical barriers (Bentzen et al., 1989; Bernatchez et al., 1992; Taylor et al., 1999; Kotlík and Berrebi, 2001; Taillebois et al., 2013). In an evolutionary context, the differentiation between populations can be significant when these factors operate, since they can limit the gene flow. In introduced populations, factors that operate during the founding and perhaps during the early generations of these populations, such as the founder effects, the limited effective population size or genetic drift, are expected to promote this type of biogeographic structure. Regardless of determining the origin of the haplotype breaks, this issue emerges as an interesting research field for future studies to gain insight into the evolutionary significance of historical introductions of the rainbow trout in Chile. This line of investigation, along with the genetic variation monitoring, could help to design more effective management actions for the maintenance of the naturalized rainbow trout populations in our country, focused on preserving potential biogeographic patterns and genetic diversity.

## Conclusion

In conclusion, our results indicate that naturalized rainbow trout populations from southern Chile present genetic structuring, a pattern that may be associated with the multiple origins of the populations, likely represented by lineages originating in different sites from western North America, such as the Kern River rainbow trout from California, the coastal rainbow trout from the Sacramento River, the steelhead trout from central California and from north of Cape Mendocino and the redband trout from the Columbia River basin. The presence of these lineages also suggests that Chilean populations may contain at least three major ecotypes, namely rainbow trout, steelhead trout and redband trout.

## Data Availability Statement

All datasets for this study are included in the article/**Supplementary Material**.

## Ethics Statement

This study was carried out in accordance with the recommendations of the Guidelines for the Use of Fishes in Research (http://fisheries.org/guide-for-the-use-offishes-in-research). The protocol was approved by the Chilean Undersecretary of Fisheries and Aquaculture (permits #2750-20-09-07 and #1580-20-05-2016).

## Author Contributions

NC, JEC and VM designed the research. Material preparation, data collection and analysis were performed by NC, FE, AA, LB, CC-A, and RC. The first draft of the manuscript was written by NC and all authors commented on previous versions of the manuscript. All authors read and approved the final manuscript.

## Funding

This study was supported by grants from Subsecretaría de Pesca y Acuicultura, Ministerio de Economía, Fomento y Turismo, Gobierno de Chile (grant 2012-34-FAP-5) and by the Dirección de Investigación, Universidad de Los Lagos (grant 0107F). The publication fee of this work was supported by the Dirección de Investigación and the Departamento de Ciencias Biológicas y Biodiversidad of the Universidad de Los Lagos.

## Conflict of Interest

Author FE was employed by company Piscícola Huililco Ltda.

The remaining authors declare that the research was conducted in the absence of any commercial or financial relationships that could be construed as a potential conflict of interest.
