# Supplementary material for: Genetic Differentiation and Origin of Naturalized Rainbow Trout Populations From Southern Chile, Revealed by the mtDNA Control Region Marker
Source: Front Genet. 2019 Dec 20;10:1212. doi: 10.3389/fgene.2019.01212 (PMC6933019; doi:10.3389/fgene.2019.01212)
Supplement: Supplementary file 1 [file Table_1.docx]

| **SUPPLEMENTARY TABLE 1** mtDNA CR reference haplotypes. The haplotypes are sorted by haplogroups and they show frequencies recorded in native populations, that include location, ecotype and code used in figures. Haplogroups are according to Bagley and Gall (1998). | | | | | | | | |
| --- | --- | --- | --- | --- | --- | --- | --- | --- |
| **Haplotype** | **Haplogroup** | **Haplotype frequency (%)** | **Populations** | **Locations** | **Ecotypes** | **Code** | **Genbank No.** | **References** |
| RTDL01 | I | 100 | Smith Creek | Nevada (USA) | Redband trout (Columbia River basin, interior) | RB-CRBI01 | AF044130 | Bagley and Gall 1998 |
| RTDL01 | I | 87.5 | Red Cow Creek | Nevada (USA) | Redband trout (Columbia River basin, interior) | RB-CRBI02 | AF044130 | Bagley and Gall 1998 |
| RTDL01 | I | 92.8 | Big Jack Creek | Nevada (USA) | Redband trout (Columbia River basin, interior) | RB-CRBI03 | AF044130 | Bagley and Gall 1998 |
| RTDL02 | I | 96.5 | Pine Creek (Pit River) | California (USA) | Redband trout (Goose Lake) | RB-GL01 | AF044131 | Bagley and Gall 1998 |
| RTDL10 | I | 100 | Davis Creek | California (USA) | Redband trout (Goose Lake) | RB-GL02 | AF044139 | Bagley and Gall 1998 |
| RTDL10 | I | 50 | Lassen Creek | California (USA) | Redband trout (Goose Lake) | RB-GL03 | AF044139 | Bagley and Gall 1998 |
| RTDL36 | I | 5 | Pine Creek (at New Pine) | California (USA) | Redband trout (Goose Lake) | RB-GL04 | AF044165 | Bagley and Gall 1998 |
| RTDL26 | I | 100 | Trout Creek | California (USA) | Redband trout (McCloud River) | RB-MR01 | AF044155 | Bagley and Gall 1998 |
| RTDL16 | II | 45.4 | Kern at Milestone Creek | California (USA) | Golden trout (Kern River Basin) | GT-KRB01 | AF044145 | Bagley and Gall 1998 |
| RTDL16 | II | 12.5 | Kern at Junction Meadow | California (USA) | Golden trout (Kern River Basin) | GT-KRB02 | AF044145 | Bagley and Gall 1998 |
| RTDL16 | II | 100 | Mears Creek | California (USA) | Rainbow trout (Sacramento River coastal) | RT-SRC01 | AF044145 | Bagley and Gall 1998 |
| RTDL16 | II | 100 | Soda Creek | California (USA) | Rainbow trout (Sacramento River coastal) | RT-SRC02 | AF044145 | Bagley and Gall 1998 |
| RTDL16 | II | 47.8 | Box Canyon, Sacramento River | California (USA) | Rainbow trout (Sacramento River coastal) | RT-SRC03 | AF044145 | Bagley and Gall 1998 |
| RTDL20 | II | 4.7 | Kern at Redspur Creek | California (USA) | Rainbow trout (Kern River) | RT-KR01 | AF044149 | Bagley and Gall 1998 |
| RTDL16 | II | 20 | Coralitos Creek | California (USA) | Steelhead (Central California) | SH-CCA01 | AF044145 | Bagley and Gall 1998 |
| RTDL22 | III | 100 | Upper Little Kern River | California (USA) | Golden trout (Little Kern River) | GT-KRB03 | AF044151 | Bagley and Gall 1998 |
| RTDL23 | III | 71.4 | Kern at Peppermint Creek | California (USA) | Rainbow trout (Kern River) | RT-KR02 | AF044152 | Bagley and Gall 1998 |
| RTDL23 | III | 86.3 | Kern at Kern Flat | California (USA) | Rainbow trout (Kern River) | RT-KR03 | AF044152 | Bagley and Gall 1998 |
| RTDL23 | III | 57.1 | Kern at Redspur Creek | California (USA) | Rainbow trout (Kern River) | RT-KR04 | AF044152 | Bagley and Gall 1998 |
| RTDL23 | III | 62.5 | Kern at Junction Meadow | California (USA) | Rainbow trout (Kern River) | RT-KR05 | AF044152 | Bagley and Gall 1998 |
| RTDL23 | III | 95.4 | Golden Trout Creek | California (USA) | Golden trout (Volcano Creek) | GT-VC01 | AF044152 | Bagley and Gall 1998 |
| RTDL23 | III | 85.7 | Johnson Creek | California (USA) | Golden trout (Volcano Creek) | GT-VC02 | AF044152 | Bagley and Gall 1998 |
| RTDL23 | III | 100 | Stokes Stringer Creek | California (USA) | Golden trout (Volcano Creek) | GT-VC03 | AF044152 | Bagley and Gall 1998 |
| RTDL07 | IV | 10 | Coralitos Creek | California (USA) | Steelhead (Central California) | SH-CCA02 | AF044136 | Bagley and Gall 1998 |
| RTDL29 | V | 13.6 | Kern at Milestone Creek | California (USA) | Rainbow trout (Kern River) | RT-KR06 | AF044158 | Bagley and Gall 1998 |
| RTDL32 | V | 71.4 | Eagle Lake Hatchery | California (USA) | Rainbow trout (Eagle Lake) | RT-EL01 | AF044161 | Bagley and Gall 1998 |
| MYS01K-1-EU | NA | 25 | Yakoun River | British Columbia (CAN) | Steelhead (North of Cape Mendocino) | SH-NCM01 | KP668855 | Stanković et al. 2016 |
| MYS01K-1-EU | NA | 28.5 | Copper River | British Columbia (CAN) | Steelhead (North of Cape Mendocino) | SH-NCM02 | KP668855 | Stanković et al. 2016 |
| MYS01K-1-EU | NA | 80 | Hoh River | Washington (USA) | Steelhead (North of Cape Mendocino) | SH-NCM03 | KP668855 | Stanković et al. 2016 |
| MYS01K-1-EU | NA | 66.6 | Hood River | Oregon (USA) | Steelhead (North of Cape Mendocino) | SH-NCM04 | KP668855 | Stanković et al. 2016 |
| MYS01K-1-EU | NA | 77.7 | Tzenzaicut Lake | British Columbia (USA) | Redband trout (Columbia River basin) | RB-CRB01 | KP668855 | Stanković et al. 2016 |
| MYS01K-1-EU | NA | 100 | West Fork Trout Creek | Washington (USA) | Redband trout (Columbia River basin) | RB-CRB02 | KP668855 | Stanković et al. 2016 |
| MYS01K-1-EU | NA | 66.6 | Fisher River | Montana (USA) | Redband trout (Columbia River basin) | RB-CRB03 | KP668855 | Stanković et al. 2016 |
| MYS01K-1-EU | NA | 56.2 | Little Sheep Creek | Oregon (USA) | Redband trout (Columbia River basin) | RB-CRB04 | KP668855 | Stanković et al. 2016 |
| MYS03C-RTDL34-EU | NA | 25 | Upper Williamson River | Oregon (USA) | Redband trout (Northern Great Basin) | RB-CRB05 | KP668864 | Stanković et al. 2016 |
| MYS03C-RTDL34-EU | NA | 20 | Witham Creek | Oregon (USA) | Redband trout (Northern Great Basin) | RB-CRB06 | KP668864 | Stanković et al. 2016 |
