# Supplementary material for: Genetic Differentiation and Origin of Naturalized Rainbow Trout Populations From Southern Chile, Revealed by the mtDNA Control Region Marker
Source: Front Genet. 2019 Dec 20;10:1212. doi: 10.3389/fgene.2019.01212 (PMC6933019; doi:10.3389/fgene.2019.01212)
Supplement: Supplementary file 2 [file Table_2.docx]

| **SUPPLEMENTARY TABLE 2** Populations studied by Bagley and Gall (1998) and Stanković et al. (2016) that contain the four haplotypes registered in the naturalized populations of rainbow trout from southern Chile. This data set includes populations where the haplotype was recorded, the locations, ecotypes, codes and the mtDNA CR haplotypes with frequencies and the assigned haplogroup, are according to Bagley and Gall (1998). | | | | | | | | |
| --- | --- | --- | --- | --- | --- | --- | --- | --- |
| **Populations** | **Locations** | **Ecotypes** | **Code** | **Haplotype** | **Haplotype frequency (%)** | **Haplogroup** | **Genbank No.** | **References** |
| Kern at Milestone Creek | California (USA) | Golden trout (Kern River Basin) | GT-KRB01 | RTDL16 | 45.4 | II | AF044145 | Bagley and Gall 1998 |
| Kern at Junction Meadow | California (USA) | Golden trout (Kern River Basin) | GT-KRB02 | RTDL16 | 12.5 | II | AF044145 | Bagley and Gall 1998 |
| Mears Creek | California (USA) | Rainbow trout (Sacramento River coastal) | RT-SRC01 | RTDL16 | 100 | II | AF044145 | Bagley and Gall 1998 |
| Soda Creek | California (USA) | Rainbow trout (Sacramento River coastal) | RT-SRC02 | RTDL16 | 100 | II | AF044145 | Bagley and Gall 1998 |
| Box Canyon, Sacramento River | California (USA) | Rainbow trout (Sacramento River coastal) | RT-SRC03 | RTDL16 | 47.8 | II | AF044145 | Bagley and Gall 1998 |
| Coralitos Creek | California (USA) | Steelhead (Central California) | SH-CCA01 | RTDL16 | 20 | II | AF044145 | Bagley and Gall 1998 |
| Kern at Redspur Creek | California (USA) | Rainbow trout (Kern River) | RT-KR01 | RTDL20 | 4.7 | II | AF044149 | Bagley and Gall 1998 |
| Yakoun River | British Columbia (CAN) | Steelhead (North of Cape Mendocino) | SH-NCM01 | MYS01K-1-EU | 25 | NA | KP668855 | Stanković et al. 2016 |
| Copper River | British Columbia (CAN) | Steelhead (North of Cape Mendocino) | SH-NCM02 | MYS01K-1-EU | 28.5 | NA | KP668855 | Stanković et al. 2016 |
| Hoh River | Washington (USA) | Steelhead (North of Cape Mendocino) | SH-NCM03 | MYS01K-1-EU | 80 | NA | KP668855 | Stanković et al. 2016 |
| Hood River | Oregon (USA) | Steelhead (North of Cape Mendocino) | SH-NCM04 | MYS01K-1-EU | 66.6 | NA | KP668855 | Stanković et al. 2016 |
| Tzenzaicut Lake | British Columbia (USA) | Redband trout (Columbia River basin) | RB-CRB01 | MYS01K-1-EU | 77.7 | NA | KP668855 | Stanković et al. 2016 |
| West Fork Trout Creek | Washington (USA) | Redband trout (Columbia River basin) | RB-CRB02 | MYS01K-1-EU | 100 | NA | KP668855 | Stanković et al. 2016 |
| Fisher River | Montana (USA) | Redband trout (Columbia River basin) | RB-CRB03 | MYS01K-1-EU | 66.6 | NA | KP668855 | Stanković et al. 2016 |
| Little Sheep Creek | Oregon (USA) | Redband trout (Columbia River basin) | RB-CRB04 | MYS01K-1-EU | 56.2 | NA | KP668855 | Stanković et al. 2016 |
| Upper Williamson River | Oregon (USA) | Redband trout (Northern Great Basin) | RB-CRB05 | MYS03C-RTDL34-EU | 25 | NA | KP668864 | Stanković et al. 2016 |
| Witham Creek | Oregon (USA) | Redband trout (Northern Great Basin) | RB-CRB06 | MYS03C-RTDL34-EU | 20 | NA | KP668864 | Stanković et al. 2016 |
